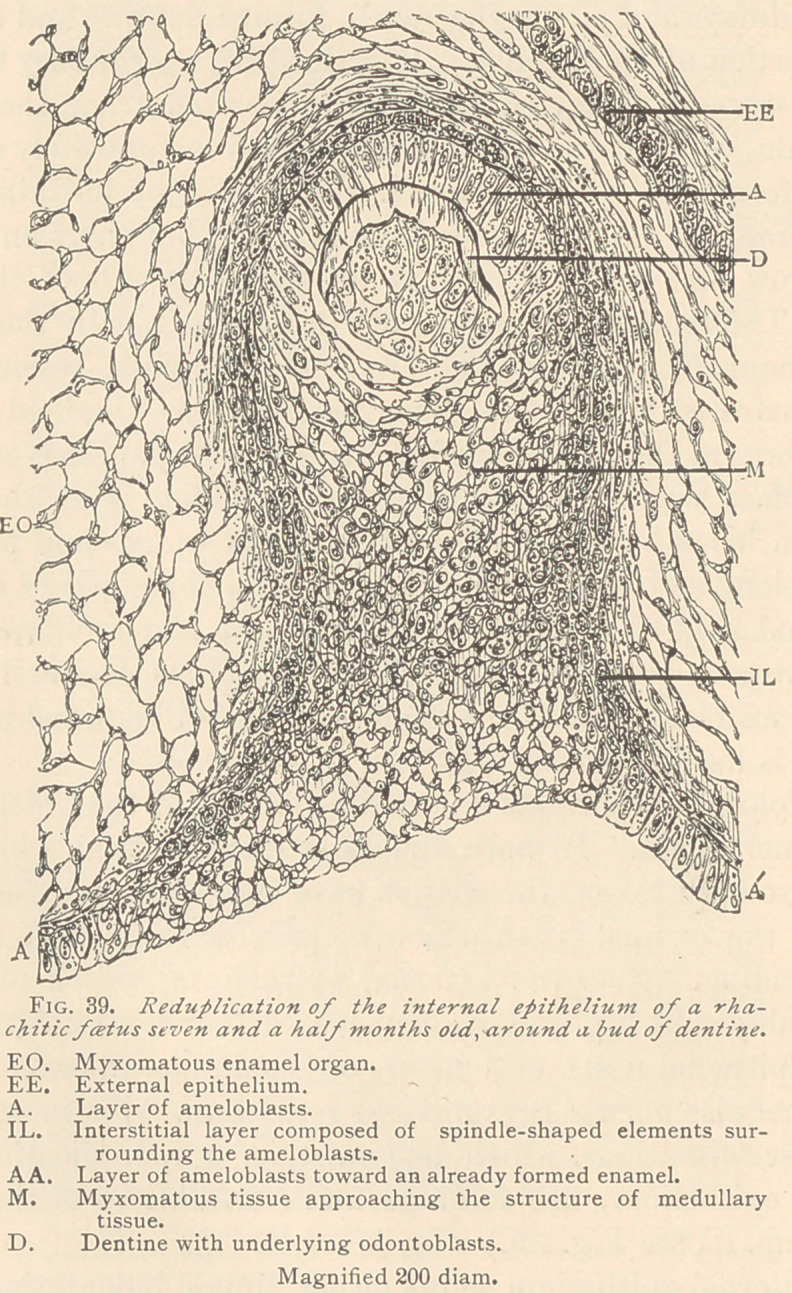# Contributions to the History of Development of the Teeth

**Published:** 1887-12

**Authors:** Carl Heitzmann, C. F. W. Bödecker


### Independent Practitioner.

VOL. VIII. DECEMBER, 1887. NO. 12.

NOTE.—No paper published or to be published in another journal will be accepted for this department. All papers must be in the hands of the Editor before the first day of the month preceding that in which they are expected to appear. Extra copies will be furnished to each contributor of an accepted original article, and reprints, in pamphlet form, may be had at the cost of the paper, press-work and binding, if ordered when the manuscript is forwarded. The Editor and Publishers are not responsible for the opinions expressed by contributors. The journal is issued promptly, on the first day of each month.

### Original Communications.

### CONTRIBUTIONS TO THE HISTORY OF DEVELOPMENT OF THE TEETH.

BY CARL HEITZMANN, M. D., AND C. F. W. BÖDECKER, D. D. S., M. D. S.

CONTINUED FROM PAGE 569.

A peculiar feature of both the papilla and the surrounding connective tissue is the presence of rusty-brown, needle-shaped crystals of hæmatoidine, sometimes clustered together in numerous masses. These are the result of a previous hæmorrhage, although it is not explicable how they appear in the papilla where the blood-vessels are extremely scanty, and just in the process of formation.

IV. Malformations and malpositions of the enamel organ. In the teeth of a rhachitic fœtus we not infrequently meet with enamel organs markedly differing from normal ones, which difference mainly consists in the lack of a myxomatous reticulum. This tissue appears in the shape of small, glistening granules, arranged either in the shape of clusters or an indistinct reticulum. The granules themselves vary somewhat in size, and among them larger granular corpuscles may be seen, which, without any regular arrangement, are yet entitled to the name of nuclei. The meshes of this irregular reticulum hold an apparently structureless mucoid

FIG. 38. *Anomalous location and formation of the enamel organ of a human fœtus seven and a half months old, affected with congenital rhachitis.*

EO. Enamel organ.

EE. External epithelium.

E. Enamel.

D. Dentine.

P. Papilla.

Magnified 100 diam.

basis-substance. Besides these irregular formations of the enamel organ, we sometimes meet with malpositions of it, either in a normal or an anomalous condition. (See Fig. 38.)

In this specimen the enamel organ is located entirely above a tooth which is likewise anomalous. It is widened in a horizontal direction, nearly parallel to the outer surface of the mucosa. It extends downward along the newly formed dentine and along the upper portion of the papilla. It is lined with medullary tissue and with clusters of epithelia, both being derived from the external epithelium, whereas the internal epithelium is completely exhausted for the formation of the enamel. The layer of medullary tissue arising from the external epithelium is broad, but short on one side, and narrow and much elongated on the other side of the enamel organ. The tooth is anomalous not in its size, nor in the stage of development, but on account of its devious papilla, resembling the teeth illustrated in Fig. 37. The specimen was obtained from the same embryo as those illustrated in Fig. 37. The papilla is constricted at the place where the dentine begins, which is at the neck of the tooth, below which the papilla suddenly widens and produces a bluntly elongated body of considerable size, composed of medullary tissue, and holding a number of clusters of hæmatoidine crystals, which are also seen in the neighboring fibrous connective tissue. Another misplacement of the enamel organ is illustrated in Fig. 36, where it is located beneath the base of the papilla.

V. Folds, convolutions and reduplications of the epithelium of the enamel organ. In both rhachitic embryo from which specimens have been taken, the writers have met with peculiar formations in the enamel organ which we propose to describe under the above heading. Near the external epithelium, which at this age is invariably split up, we have sometimes found concentrically arranged epithelial nests, or clusters of medullary tissue, imbedded in an otherwise normal myxomatous reticulum. Far more common than these formations, which depend upon a reduplication of the external epithelium, are foldings and convolutions of the internal epithelium. (See Fig. 39.)

The internal epithelium shows either simple indentations, which are sometimes also observed in normal teeth of the same stage of development. At other times there is a series of successive convolutions of the internal epithelium in whose neighborhood the myxomatous tissue of the enamel organ, as a rule, is in a medullary condition, which we found with and without a pronounced intermediate layer. Obviously, such sinuosities correspond to furrows of the enamel organ, and very probably may cause the ridges and furrows often observed upon this tissue. The highest degree of reduplication of the internal epithelium is sometimes seen together with apparently isolated buds of dentine, surrounded by and inclosed in a layer of ameloblasts and myxomatous tissue. The dentine consists either of a narrow calcareous rim, or a cap in which we can observe distinct dentinal canaliculi. If the dentinal cap appears as a thin calcified ledge, it is composed of calcified medullary corpuscles, beneath which odontoblasts may be observed. If the ledge of the dentine is broader and supplied with dentinal canaliculi, we notice close beneath it a layer of medullary corpuscles followed by a layer of odontoblasts, which usually are in the process of breaking up into medullary corpuscles. It may be possible that such formations are the starting points of the transverse furrows observed upon the labial surfaces of the temporary teeth of rickety children, although these occurrences are far more common on permanent teeth.

(TO BE CONTINUED.)

## Figures and Tables

**Fig. 38. f1:**
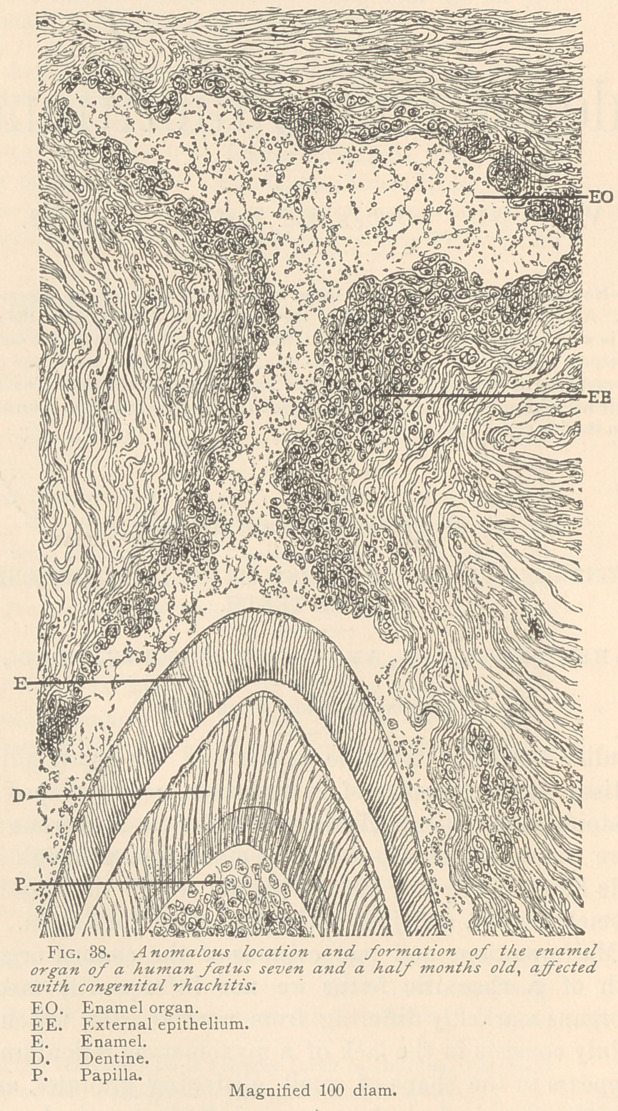


**Fig. 39. f2:**